# Methods to evaluate the performance of a multicomponent meningococcal serogroup B vaccine

**DOI:** 10.1128/msphere.00898-24

**Published:** 2025-04-08

**Authors:** Ray Borrow, Laura Tomasi Cont, Daniela Toneatto, Stefania Bambini, Shravani Bobde, Woo-Yun Sohn, Alessia Biolchi, Vega Masignani, Peter T. Beernink, Maria Lattanzi

**Affiliations:** 1 Meningococcal Reference Unit, UK Health Security Agency, Manchester Royal Infirmary, Manchester, United Kingdom; 2 GSK, Siena, Italy; 3 GSK, Rockville, Maryland, USA; 4 University of California, San Francisco, California, USA; University of Michigan-Ann Arbor, Ann Arbor, Michigan, USA

**Keywords:** 4CMenB, invasive meningococcal disease, *Neisseria meningitidis*, serum bactericidal antibody assay, vaccine, vaccine effectiveness

## Abstract

Meningococcal serogroup B (MenB) vaccine licensure was based on the assessment of vaccine-induced immune responses by human serum bactericidal antibody (hSBA) assay against a small number of antigen-specific strains complemented by strain coverage predictions. However, the evaluation of vaccine strain coverage is challenging because of genotypic and phenotypic diversity in surface-exposed MenB strain antigens. This narrative review considers the principal methods applied to assess the performance of a multicomponent MenB vaccine at different stages of its development. Traditional hSBA assay against a limited panel of strains is useful at all stages, while predicted strain coverage methods, such as the meningococcal antigen typing system, are used independent of clinical trials. A new method, the endogenous complement hSBA assay, has been developed to evaluate a vaccine’s ability to induce a bactericidal immune response in clinical trials, in conditions that approximate real-world settings through the use of each vaccinee’s serum as a source of complement and by testing against a panel of 110 epidemiologically representative MenB strains. Each assay, therefore, has a different scope during the vaccine’s development and all complement each other, enabling comprehensive evaluation of the performance of multicomponent MenB vaccines, in advance of real-world evidence of vaccine effectiveness and vaccine impact.

## INTRODUCTION

Invasive meningococcal disease (IMD), caused by *Neisseria meningitidis* and manifesting most often as meningitis and/or septicemia, is life-threatening and unpredictable, with diverse and serious sequelae experienced at high incidences by survivors (1–3). For many other vaccine-preventable diseases, phase 3 randomized clinical trials provide critical data for licensure on vaccine efficacy, defined as the percentage reduction in disease incidence in a vaccinated versus a non-vaccinated population (4, 5), and safety. However, it is not feasible to determine vaccine efficacy against IMD in trials because its low incidence would require impractically large numbers of individuals to be enrolled (4). Evidence for the licensure of meningococcal vaccines was therefore based on the accepted serological surrogate measure of protection for meningococcal vaccines, i.e., the immunological response established by serum bactericidal antibody (SBA) assay using an exogenous source of human complement (hSBA assay) against “test” or “indicator” strains identified to assess antigen-specific killing induced by antibodies elicited by each vaccine component (6, 7). This does not provide information on cross-protection versus diverse circulating strains with antigen variants that differ from those in the vaccine or have lower expression levels.

Meningococcal serogroups A, B, C, W, and Y cause most cases of IMD worldwide (8, 9), and effective vaccines are available against serogroups A, C, W, and Y (MenACWY) and serogroup B (MenB) (10). While MenACWY vaccines are polysaccharide-protein conjugated vaccines, poor immunogenicity of the MenB capsular polysaccharide (because of structural similarity with a surface-exposed polysaccharide present on the human neural cell adhesion molecule) and the potential to induce autoimmune antibodies prompted the development of noncapsular, protein-based MenB vaccines (10, 11). Licensure of 4CMenB (Bexsero, GSK) and MenB-FHbp (Trumenba, Pfizer) was based on immunogenicity assessment by hSBA assay complemented by strain coverage predictions (5). In the United States, both vaccines are licensed for persons aged 10–25 years (12); in Europe and other regions, 4CMenB is licensed for those aged 2 months or older (13) and MenB-FHbp for individuals aged 10 years and older (14). 4CMenB is a multicomponent MenB vaccine with four antigenic components: factor H binding protein (fHbp; peptide 1.1), *Neisseria* adhesin A (NadA; peptide 3.8), neisserial heparin-binding antigen (NHBA; peptide 2), and outer membrane vesicles (OMVs) containing porin A (PorA; subtype P1.4) protein as the immunodominant antigen (13, 15). These antigens, which are conserved to various extents and induce a robust bactericidal immune response across MenB strains (13, 16), were selected from the MenB whole genome sequence by reverse vaccinology, the genome to vaccine “bottom-up” development approach (15, 17). The rationale for combining four different antigens was to increase the spectrum of vaccine strain coverage and minimize the risk of bacterial immune evasion due to antigen mutation or loss (18). MenB-FHbp contains two variants of fHbp (peptides 3.45 and 1.55, corresponding to variants A05 and B01) (14). A pentavalent MenABCWY vaccine (Penmenvy, GSK) that combines components of 4CMenB and the licensed meningococcal ACWY CRM_197_-glycoconjugate vaccine (MenACWY-CRM; Menveo, GSK) was approved by the U.S. Food and Drug Administration (FDA) for use in individuals aged 10–25 years in February 2025 (19, 20), and a MenABCWY vaccine (Penbraya, Pfizer) containing components of MenB-FHbp and MenACWY tetanus toxoid conjugate vaccine (MenACWY-TT; Nimenrix, Pfizer) was approved for the same age group in October 2023 (21).

The efficacy of the MenACWY component of the pentavalent vaccines can be inferred by assessing serogroup-specific anti-capsular antibodies that elicit bactericidal activity (22). This is because capsular polysaccharides are extremely well conserved among strains of the same serogroup and abundant, so hSBA against a single serogroup-specific test strain is sufficient to confirm coverage against all strains belonging to that serogroup. The same does not apply to the MenB component. While circulating MenB strains also have conserved capsular polysaccharides, surface protein antigens display variability in amino acid sequences and protein expression levels (5, 23). Consequently, the effectiveness of noncapsular protein-based MenB vaccines may vary in real-world settings.

**Fig 1:**
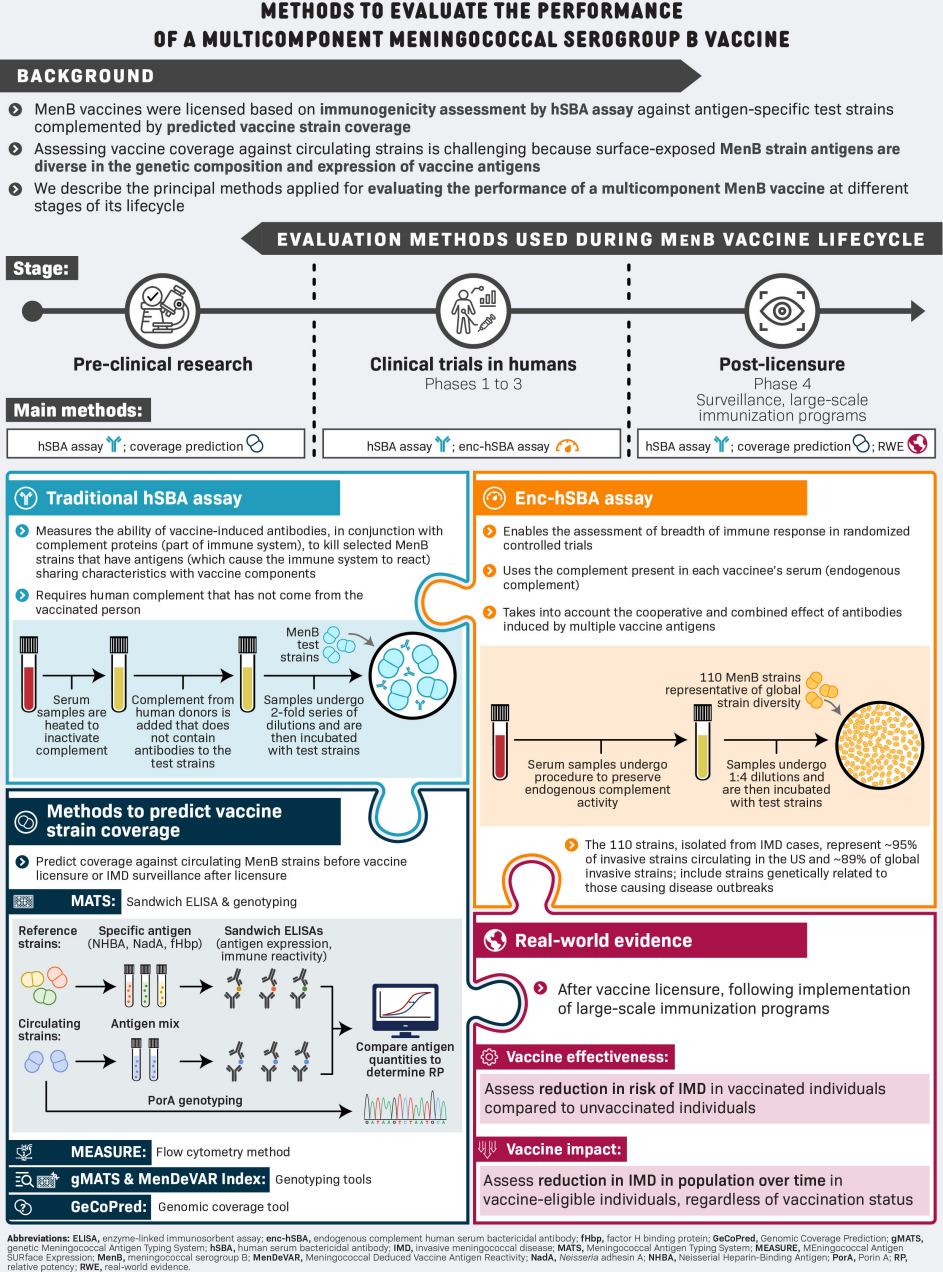
Summary of methods to evaluate the performance of multicomponent meningococcal serogroup B (MenB) vaccines, i.e., vaccines that can elicit bactericidal antibodies against their different antigenic components.

Assessment of the strain coverage of multicomponent MenB vaccines, i.e., vaccines that can elicit bactericidal antibodies against their different immunogenic components, requires a reliable means of determining the susceptibility of circulating MenB strains to killing by vaccine-elicited antibodies (16). In this narrative review, we describe the principal methods applied during vaccine development to evaluate vaccine-induced immune responses and MenB strain coverage and highlight a new assay of multicomponent MenB vaccine performance in clinical trials, the endogenous complement hSBA (enc-hSBA) assay, conducted under conditions close to real-world settings. A summary is provided in Fig. 1.

## PRINCIPAL METHODS USED TO ASSESS MenB VACCINE PERFORMANCE

**Fig 2:**
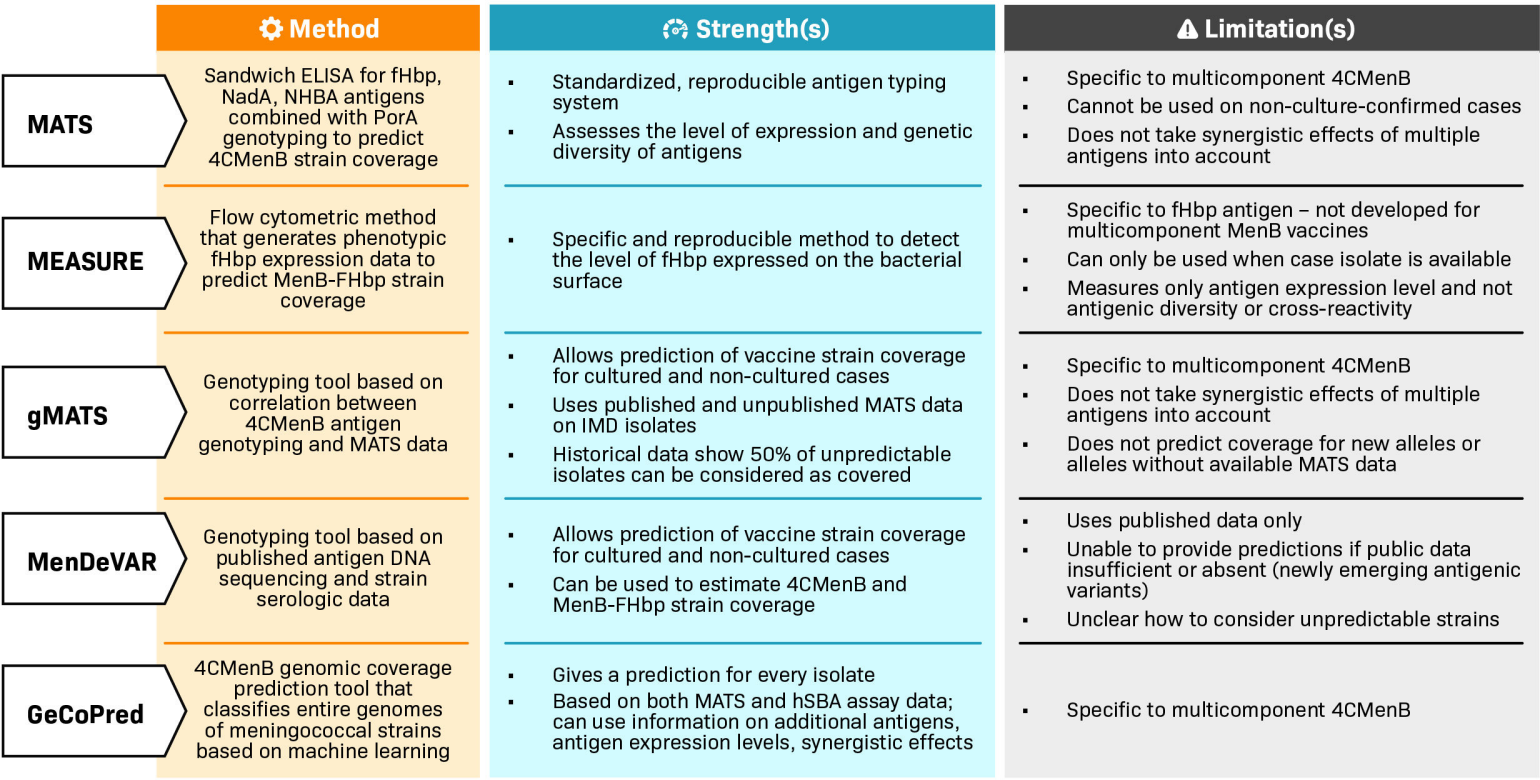
Methods to predict meningococcal serogroup B (MenB) vaccine strain coverage: meningococcal antigen typing system (MATS) (24, 25), meningococcal antigen surface expression (MEASURE) assay (26), genetic MATS (gMATS) (27), meningococcal deduced vaccine antigen reactivity (MenDeVAR) index (28), and genomic coverage prediction (GeCoPred) (29). 4CMenB, 4-component meningococcal serogroup B vaccine; ELISA, enzyme-linked immunosorbent assay; fHbp, factor H binding protein; hSBA, human serum bactericidal antibody; IMD, invasive meningococcal disease; MenB-FHbp, bivalent factor H binding protein meningococcal serogroup B vaccine; NadA, *Neisseria* adhesin A; NHBA, neisserial heparin-binding antigen; PorA, porin A.

The main method for evaluating the performance of multicomponent MenB vaccines is the hSBA assay in clinical trial settings (Fig. S1), while outside of clinical trials, different methods are used to predict the coverage of MenB vaccines against representative collections of circulating meningococcal strains (Fig. 2).

The traditional hSBA assay measures complement-mediated bacterial killing (30). It originated in the 1960s from an analysis by Goldschneider and colleagues of serum from military recruits at a U.S. Army base during an outbreak of invasive serogroup C disease (31). Comparing healthy recruits with those with IMD, the minimum functional antibody titer associated with protection was 4 (i.e., ≥1:4 serum dilution).

In the traditional hSBA assay of MenB vaccines (Fig. S1), serum samples, as the sources of antibody, are heated to inactivate intrinsic complement proteins, and standardized exogenous human complement is added to measure bactericidal antibody responses in the samples (32). This includes antibody geometric mean titers (GMTs) and clinical endpoint measures, such as percentage of participants with hSBA titers above the cut-off regarded as indicative of seroprotection and the proportion of vaccinees with fourfold rises or more in hSBA titer pre-vaccination to post-vaccination (33). For serum samples from individuals vaccinated with 4CMenB or the pentavalent vaccine containing 4CMenB, immunogenicity was assessed against four indicator strains with susceptibility to killing mediated by antibodies mainly directed against one of the four principal antigens present in 4CMenB (fHbp, NadA, NHBA, or PorA) (15, 34–36). This allows the contribution of each vaccine antigen in generating a specific immune response to be evaluated. MenB-FHbp immunogenicity has been assessed against a random selection of strains harboring vaccine-heterologous fHbp variants that were representative of the diversity of MenB isolates, with low to medium fHbp surface expression (37): four primary strains, which express fHbp variants representing 42% of MenB isolates in a panel from the United States and Europe, and 10 additional strains expressing fHbp variants representing an additional 39% of isolates in the same panel. The immunogenicity of the MenB component of the MenABCWY vaccine containing MenB-FHbp and MenACWY-TT was assessed against the four primary strains only (38).

Although generally accepted by regulatory authorities as providing an estimate of vaccine protection, endpoints determined by hSBA assay do not represent the performance of MenB vaccines against real-world, diverse circulating strains. This assay can only be performed against a limited number of strains because the exogenous complement must be seronegative for anti-meningococcal antibodies for each tested strain, requiring laborious screening of complement from healthy unvaccinated individuals (39). Immunoglobulin G and M antibody-depleted serum could conceivably be used as a source of complement (40), but this would need to be obtained in a sufficient quantity to perform thousands of assays. Also, a large amount of serum (as an antibody source) would have to be obtained from vaccinated individuals, which may be especially challenging in the case of young children.

Methods for predicting MenB vaccine strain coverage are useful for initial assessments within a given country or region before vaccine licensure, and before real-world efficacy data are generated from immunization programs, and for meningococcal disease surveillance after vaccine licensure (Fig. 2).

The meningococcal antigen typing system (MATS) was developed to predict 4CMenB strain coverage, combining a vaccine antigen-specific sandwich enzyme-linked immunosorbent assay for antigens fHbp, NadA, and NHBA with PorA genotyping information (24, 25). MATS requires cultured strains, but culture-confirmed IMD strains are not always available, usually because of early antibiotic treatment. To overcome this, the genetic MATS (gMATS) was developed (27), which was shown to mirror MATS in predicting strain coverage in an analysis of 4CMenB strain coverage against a panel of over 3,000 invasive MenB isolates from 13 countries (27). 4CMenB strain coverage point estimates were 66%–91% by MATS against epidemiologically relevant MenB strain panels from Europe, North and South America, and Australia (25, 27, 41–54), and 58%–89% by gMATS against strain panels from Europe, North America, and Australia (27, 44, 51, 53, 55, 56).

However, predicted 4CMenB strain coverage is likely to be underestimated with MATS (and gMATS) (5, 25, 57) because it does not measure the synergistic effect of antibodies recognizing different antigens (58), it underestimates the contribution of NadA to protection (59), and it does not measure the contribution of OMV components other than the presence of a matched PorA antigen (60). Indeed, OMV components porin B (PorB) and class 5 outer membrane protein (OpcA) have been identified as additional bactericidal 4CMenB antigens (61). Underestimation of strain coverage by MATS has been demonstrated (57, 62, 63); for example, the 4CMenB strain coverage estimate against a panel of 40 invasive strains isolated in England and Wales was 66%–73% by MATS and 72%–73% by gMATS, lower than the hSBA assay estimate of bacterial killing (88%) against the same panel (27, 57).

Other methods that have been developed to predict MenB vaccine strain coverage include the meningococcal antigen surface expression (MEASURE) assay (26) and meningococcal deduced vaccine antigen reactivity (MenDeVAR) index (28) (Fig. 2). MEASURE is a flow cytometric method that uses a cross-reactive monoclonal antibody to quantify the level of fHbp expressed on the MenB strain surface (fHbp expression can vary up to 15-fold among MenB strains) (64), with no expression reported in some cases (65), generating phenotypic fHbp expression data without considering protein sequence variability (26). MEASURE, therefore, determines antigen expression but not antigenic diversity since the monoclonal antibody targets a highly conserved fHbp epitope (5). Another limitation is that the MEASURE assay can only be used when a case isolate is available.

The MenDeVAR index uses antigen DNA sequencing data combined with serological characterization results to categorize MenB strains according to antigens that are matched or cross-reactive to the vaccine antigens (28). For 4CMenB strain coverage estimations, antigen genotyping is combined with MATS data, while for MenB-FHbp strain coverage estimations, antigen genotyping is combined with MEASURE and hSBA assay data. Like gMATS, the MenDeVAR index can be applied to any MenB isolate, but it is limited to antigens that are already present in public databases, so it cannot provide predictions if the data are insufficient or absent for newly emerging antigenic variants, which can create challenges for panels with high numbers of strains that are considered unpredictable because of insufficient data. With gMATS, historical data show 50% of unpredictable strains can be considered as covered (27), but for the MenDeVAR index, the appropriate approach toward unpredictable strains is unclear (66, 67).

A new tool for 4CMenB genomic coverage prediction classifies the entire genomes of *N. meningitidis* strains based on machine learning (29). This method provides a prediction for every isolate, is based on both MATS and hSBA assay data, and can use data on additional OMV components, antigen expression levels, and synergistic effects to further improve strain coverage predictions. Results are awaited from future studies of vaccine strain coverage using this method.

## HOW TO EVALUATE THE PERFORMANCE OF A MULTICOMPONENT MenB VACCINE WITH AN hSBA ASSAY: DEVELOPMENT OF THE enc-hSBA ASSAY TO ASSESS BREADTH OF IMMUNE RESPONSE IN CLINICAL STUDIES

The effectiveness and safety of MenB vaccines can only be confirmed via population-level, real-world evidence, as demonstrated for 4CMenB in various countries in the last decade (68–73). Evidence from immunization programs, comparing vaccinated and unvaccinated individuals and the same population before and after vaccination, confirms the vaccine effectiveness (VE) and vaccine impact of 4CMenB against MenB IMD (68, 69, 71, 72).

For novel vaccine formulations against endemic disease-causing MenB strains, it is increasingly important that vaccine performance is investigated in pre-licensure clinical studies. This recognition led to the development of hSBA assays that use endogenous complement present in each vaccinated person’s serum: the enc-hSBA assay against 110 diverse MenB strains, developed by GSK (74, 75) and the intrinsic complement hSBA (iSBA) assay against 30 MenB strains, developed by the U.S. FDA (76). For both assays, endogenous complement activity within each serum sample is preserved, maintaining subject-to-subject variability in complement activity. This approach is limited by the large number of tests needed to cover sufficient strains in the MenB strain panel and the inability to use it for individuals with complement deficiencies or those receiving treatments that impact the complement system (74). However, a major advantage is the ability to assess vaccine-induced antibody responses against antigenically diverse invasive disease isolates in clinical trial settings.

With the enc-hSBA assay, sera are diluted 1:4, the dilution generally considered to be linked to protection against IMD (7, 31, 32, 77), and tested against a broad panel of 110 strains that represent the overall genetic landscape of MenB strains that might be encountered globally (74, 78) (Fig. S1). The 110 MenB strains used in this assay were selected randomly from 442 isolates collected in 2000–2008 in 10 U.S. districts by the Centers for Disease Control and Prevention (CDC) Active Bacterial Core surveillance (ABCs) system (79, 80). Its repertoire of antigen genotypes is representative of different geographic regions, specifically, approximately 95% of invasive strains circulating in the United States and overall 89% of invasive strains in the United States, Canada, Europe, and Australia (78). The panel is representative of MenB strain diversity over time (81), and the 110 strains, isolated from IMD cases, include strains genetically related to clones causing IMD outbreaks (78).

By using endogenous complement and testing a diverse, epidemiologically relevant panel of MenB strains, the enc-hSBA assay evaluates the ability of the vaccine to induce bactericidal immune responses in conditions that are close to real-world settings. As such, this assay is considered capable of evaluating the breadth of immune response and the vaccine’s ability to induce a bactericidal immune response against a broad panel of MenB strains in randomized clinical trials. This takes into account strain killing potentially due to the synergistic effect of the entire repertoire of antibodies induced by more than one of the antigens included in the multicomponent vaccine (58). This cooperative effect cannot be fully described using the traditional hSBA assay that uses selected vaccine antigen-specific indicator strains, with single antigen recognition as the main basis for immunogenicity assessment. MenB strains circulating worldwide are likely to express on their surfaces one or more 4CMenB antigens that may be cross-reactive, have different levels of expression, and have antigenic sequence differences, so various combinations of vaccine protein variants in circulating strains could result in different bactericidal responses versus those obtained using indicator strains (58).

The enc-hSBA assay was qualified using the 110 MenB strain panel and further validated using the four antigen-specific indicator strains (74). It is, to date, the most suitable bactericidal assay to estimate the breadth of immune response against diverse MenB strains for vaccines containing multiple MenB components (74), and it has been used in phase 2 and 3 studies (35, 36, 75). A recent pivotal phase 3 study of 4CMenB and the investigational pentavalent MenABCWY vaccine containing 4CMenB and MenACWY-CRM antigens, in adolescents and young adults aged 10–25 years (ClinicalTrials.gov, NCT04502693), included breadth of immune response endpoints assessed by enc-hSBA assay as well as immunogenicity endpoints assessed by traditional hSBA assay (35, 36). As illustrated in Fig. S2, there were two breadth of immune response endpoints:

- test-based breadth of the immune response, based on the percentage of samples lacking bactericidal activity against the 110 MenB strain panel in the study group receiving the multicomponent MenB vaccine (MenABCWY or 4CMenB) versus the percentage in the study group receiving MenACWY-CRM, which is expected to provide no protection against MenB strains, and
- responder-based breadth of immune response, based on the proportion of vaccinated individuals whose sera killed at least 70% of strains tested from the 110 MenB strain panel, thereby estimating the percentage of study participants who mount a killing response against the majority of tested strains.

These endpoints provide measures of breadth of immune response from two perspectives—the percentage of samples with bactericidal activity against MenB strains and the proportion of individuals with sera that kill the majority of tested MenB strains—thus delivering a comprehensive assessment via enc-hSBA assay of the 110 MenB strain panel. In the phase 3 trial, test-based breadth of immune response was 78.7% (97.5% confidence interval [CI]: 77.2–80.1) and responder-based breadth of immune response was 84.8% (97.5% CI: 81.8–87.5) following two 4CMenB doses given 2 months apart, 81.8% (95% CI: 80.4–83.1) and 89.8% (95% CI: 87.2–92.0), respectively, following two 4CMenB doses 6 months apart, and 77.9% (95% CI: 76.6–79.2) and 84.1% (95% CI: 81.4–86.5), respectively, following two MenABCWY doses 6 months apart (35, 36). enc-hSBA assay data on bactericidal serum activity also showed the MenABCWY schedule was non-inferior to the 4CMenB 0–2 months schedule.

**Fig 3:**
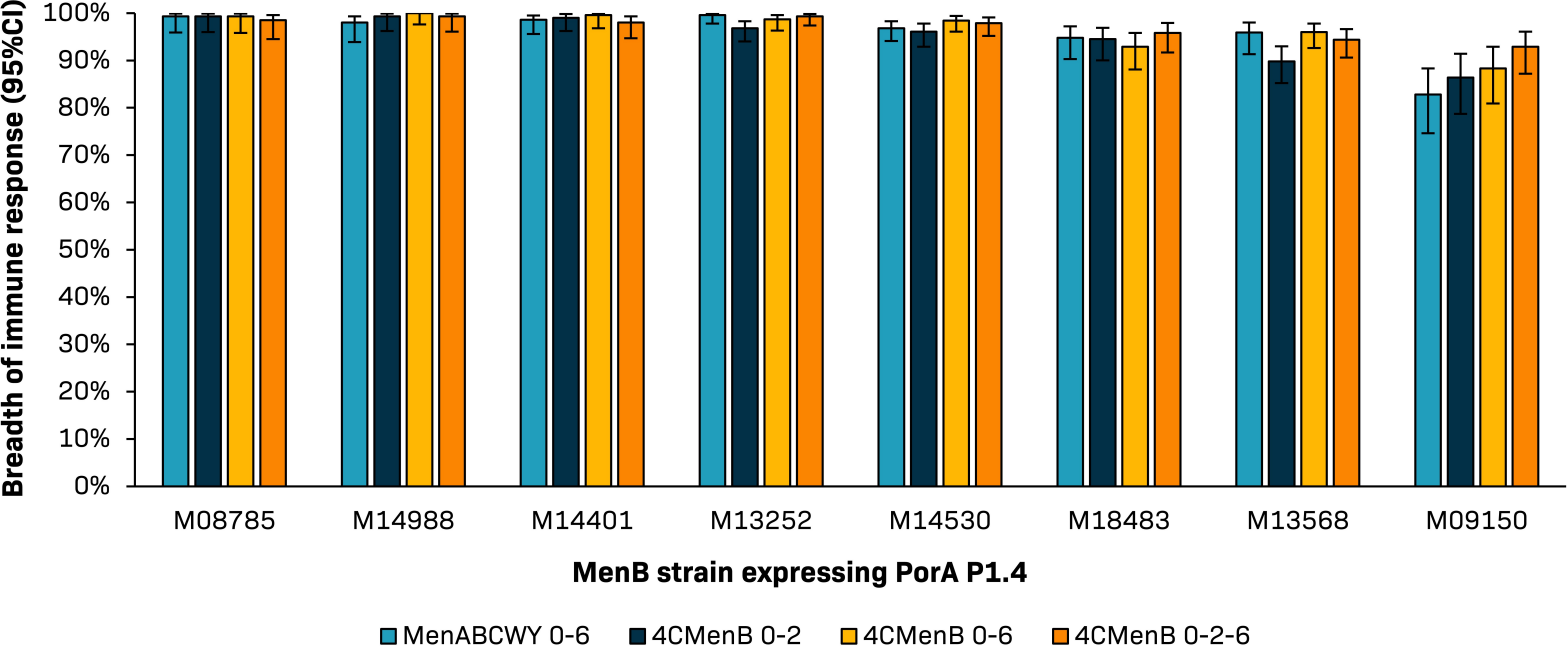
Comparable test-based breadth of immune response for investigational MenABCWY vaccine (0–6 months schedule) and 4CMenB vaccine (0–2, 0–6, and 0–2–6 months schedules), as shown by endogenous complement human serum bactericidal antibody assay against eight circulating strains from the 110 meningococcal serogroup B (MenB) strain panel that express porin A subtype P1.4 (PorA P1.4) (35). Strain M08785 has the same antigenic features as the PorA P1.4 indicator strain MenABCWY group, administered meningococcal serogroups ABCWY vaccine at months 0 and 6; 4CMenB 0–2 group, administered two doses of 4-component meningococcal serogroup B (4CMenB) vaccine at months 0 and 2; 4CMenB 0–6 group, administered two doses of 4CMenB at months 0 and 6; 4CMenB 0–2–6 group, administered three doses of 4CMenB at months 0, 2, and 6. CI, confidence interval.

Other data on the breadth of immune response from this trial are supportive of a synergistic effect of antibodies induced by the MenB component of the MenABCWY vaccine and by 4CMenB. The pre-defined criterion for non-inferiority of the MenABCWY 0–6 months schedule to the 4CMenB 0–2 months schedule, as assessed by traditional hSBA assay, was met for three of the four MenB indicator strains but not PorA because of lower hSBA titers against this indicator strain (35). PorA is a surface-exposed antigen that is highly variable across MenB strains (82, 83) and 4CMenB contains PorA serosubtype P1.4 from a New Zealand outbreak strain (NZ98/254), which is expressed by 8 of the 110 MenB strains. The clinical relevance of not meeting the success criterion for PorA is unknown. However, the enc-hSBA assay against the eight MenB circulating strains that express PorA P1.4, of which one has the same vaccine antigen variants and belongs to the same clonal complex as the PorA indicator strain, showed that the test-based breadth of immune response was comparable between the MenABCWY and 4CMenB vaccine groups for all eight strains (83%–100% versus 86%–100%) (Fig. 3) (35). This could indicate that, even with lower hSBA titers against PorA, antibodies elicited by both multicomponent MenB vaccines can be sufficient to kill PorA P1.4-matched MenB strains, as shown by the enc-hSBA assay.

## BREADTH OF IMMUNE RESPONSE BY enc-hSBA ASSAY AND REAL-WORLD EVIDENCE OF VE

Real-world evidence of the effectiveness and impact of 4CMenB has accumulated since the vaccine was first licensed in 2013, with results published in conjunction with 4CMenB mass immunization programs in various settings in the United States, Canada, Italy, Portugal, Spain, United Kingdom, and South Australia (71, 73, 84–95). With different dose schedules in different age groups (infants, toddlers, adolescents, and adults), decreases of 55%–96% in MenB IMD (71, 90–95) and VE against MenB IMD of 71%–100% (84–89, 96) have been reported.

Clinical trial data produced with the enc-hSBA assay for 4CMenB are in line with 4CMenB effectiveness data generated so far in real-world settings. Specifically, test-based and responder-based breadth of immune response point values (78.7% and 84.8%, respectively) in the phase 3 trial of adolescents and young adults who received the 4CMenB 0–2 months schedule (36) are consistent with real-world evidence of VE available from South Australia, 2 and 3 years after the two-dose 4CMenB immunization program in adolescents that ran from February 2019 to January 2021 (89, 93). This showed a 71% (95% CI: 15–90; *P* = 0.02) reduction in MenB disease cases when comparing the previous 14-year period with the 2 years after 4CMenB vaccination (93), and a 78.5% (95% CI: 33.0–93.1) reduction in MenB IMD cases and 89.4% (95% CI: 0–99.0; case-control analysis) VE against MenB disease in the 3 years after program implementation (89).

Moreover, in the phase 3 study, point values for breadth of immune response against the 110 MenB strain panel following two MenABCWY doses given 6 months apart (test-based, 77.9%; responder-based, 84.1%) were in line with those following the 4CMenB 0–2 months and 0–6 months schedules, and the MenABCWY vaccine had demonstrated non-inferiority to the 4CMenB 0–2 schedule, based on percentages of samples with bactericidal activity against the strain panel by enc-hSBA assay (35). This suggests that the investigational MenABCWY vaccine could potentially provide broad protection against MenB invasive disease, as demonstrated with 4CMenB through real-world evidence.

## enc-hSBA ASSAY COMPLEMENTS TRADITIONAL hSBA AND VACCINE STRAIN COVERAGE ASSAYS

Methods that evaluate the performance of multicomponent MenB vaccines have different applications during the vaccine development process. The enc-hSBA assay is designed to assess the breadth of immune response against MenB strains in randomized controlled clinical trials of multicomponent MenB vaccines. The test-based and responder-based measurements by enc-hSBA assay can be used to complement immunogenicity results obtained with the traditional hSBA assay, enabling a more complete assessment of the performance of multicomponent MenB vaccines in clinical trials. Additionally, results by enc-hSBA assay can complement those generated via vaccine strain coverage tools, such as MATS, gMATS, and the MenDeVAR index, as used by national reference laboratories for predicting 4CMenB strain coverage or for IMD surveillance.

Overall, the enc-hSBA assay provides an assessment of the ability of a multicomponent MenB vaccine to induce a bactericidal immune response against broad panels of epidemiologically representative MenB strains in clinical trials, in conditions close to real-world settings. With the accumulation of further enc-hSBA assay results, it will be possible to determine its role as a proxy measure of real-world VE through its combined assessment of the human immune response against MenB and predicted strain coverage.

## CONCLUSIONS: WHEN TO USE WHAT?

The overall effectiveness of a multicomponent MenB vaccine is dependent on the proportion of circulating, disease-causing MenB strains that are susceptible to antibody-mediated, complement-dependent SBA activity elicited by vaccine components. Therefore, in advance of real-world evidence of VE and vaccine impact from large-scale vaccine immunization programs, the performance of multicomponent MenB vaccines against a variety of strains can be captured using an assay that, *in vitro*, considers the synergistic effect of endogenous immune responses elicited by different vaccine antigens.

Different methods are available at different stages in the development of multicomponent MenB vaccines to assess performance. The traditional hSBA assay on MenB indicator strains is the established *in vitro* method of measuring vaccine-induced immune responses from the early phases of meningococcal vaccine development onwards and is regarded as a surrogate measure of vaccine efficacy. MATS, gMATS, MEASURE, and the MenDeVAR index are generally used for predicting MenB vaccine strain coverage on epidemiologically relevant collections of MenB strains before vaccine licensure and for meningococcal disease surveillance purposes after vaccine licensure.

The enc-hSBA assay and the similar iSBA assay, presented by the U.S. FDA at a recent international congress (76), are practical methods for assessing vaccine-induced antibody responses in each vaccinated individual against multiple diverse IMD-causing isolates, without the need to identify an external complement source for each strain. The enc-hSBA assay is a highly standardized method that allows reproducible measurement of bactericidal killing against a wide variety of circulating MenB strains representative of global strain diversity, so it gauges the vaccine’s ability to induce a bactericidal immune response in randomized clinical trials under conditions that approximate real-world settings. The enc-hSBA assay is not intended to replace MATS and gMATS in routine surveillance conducted by meningococcal reference laboratories and cannot fully replace the traditional hSBA assay as it does not allow statistical evaluation of antibody GMTs or changes in hSBA titers over time. However, the breadth of immune response for 4CMenB (recently assessed in a pivotal phase 3 trial to support its full licensure in the United States) is in line with real-world 4CMenB VE data, suggesting this measure has utility in supplementing immunogenicity results obtained with the traditional hSBA assay and strain coverage predictions. Statistical correlations between enc-hSBA assay results. hSBA assay seropositivity, and estimated strain coverage will be investigated further in future studies.

In conclusion, each method for evaluating multicomponent MenB vaccines has a different scope during the vaccine’s development. The methods complement each other, enabling comprehensive evaluations of vaccine performance in advance of real-world evidence of VE and allowing the changing epidemiology of meningococcal disease to be monitored.

## Data Availability

Data sharing is not applicable to this article as no data sets were generated or analyzed during the current study.

## SUPPLEMENTAL MATERIAL

Supplemental Material msphere.00898-24-s0001.docx Figures S1 and S2.

ASM does not own the copyrights to Supplemental Material that may be linked to, or accessed through, an article. The authors have granted ASM a non-exclusive, world-wide license to publish the Supplemental Material files. Please contact the corresponding author directly for reuse.
