# Supplementary material for: Methods to evaluate the performance of a multicomponent meningococcal serogroup B vaccine
Source: mSphere. 2025 Apr 8;10(4):e00898-24. doi: 10.1128/msphere.00898-24 (PMC12039234; doi:10.1128/msphere.00898-24)
Supplement: Supplemental Material — Figures S1 and S2. [file msphere.00898-24-s0001.docx]

## **SUPPLEMENTAL MATERIAL**

## Fig. S1. Features of the traditional human serum bactericidal antibody (hSBA) assay^1^ and the endogenous complement hSBA (enc-hSBA) assay^2^


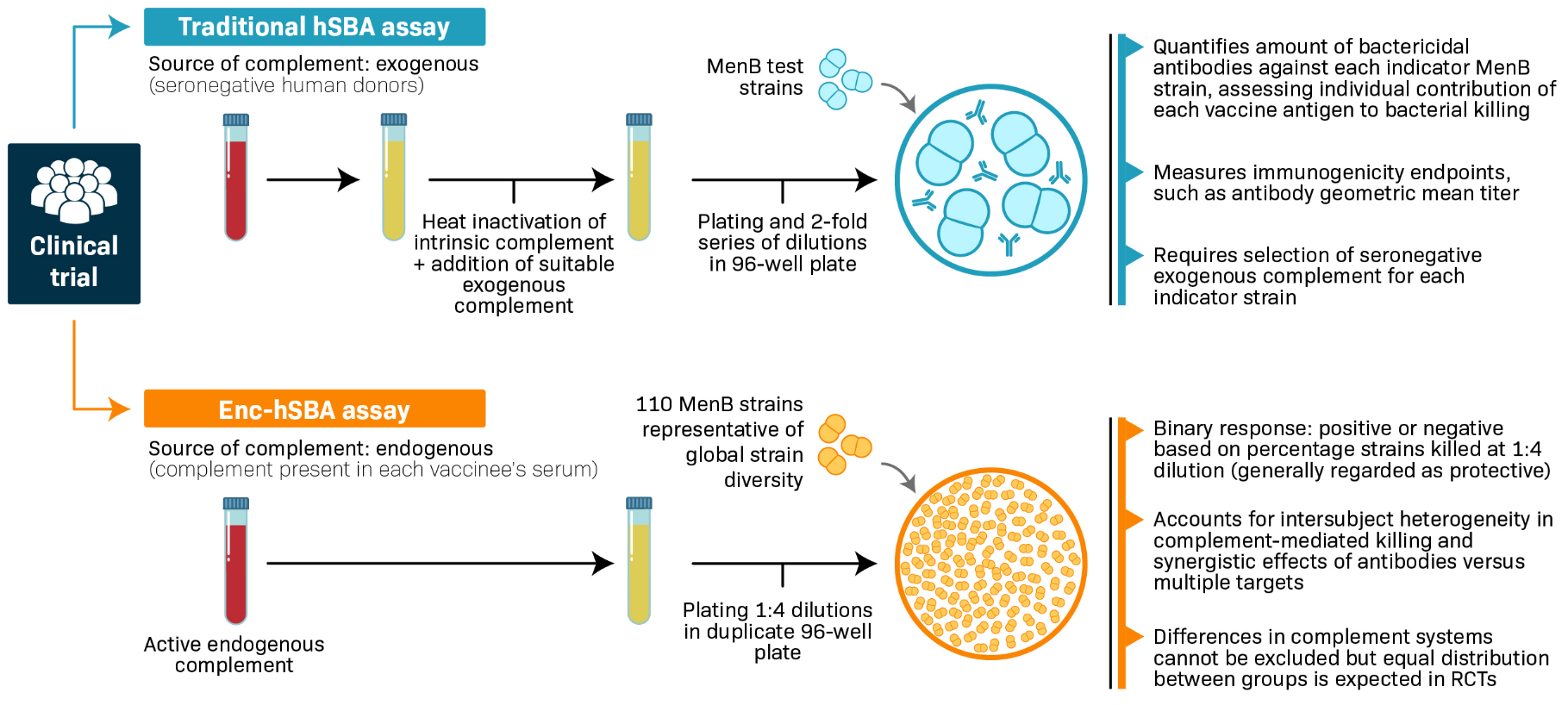


MenB, meningococcal serogroup B; RCT, randomized clinical trial

## Fig. S2. Test-based and responder-based measurement of breadth of immune response against 110 MenB strain panel for a multicomponent meningococcal serogroup B (MenB) vaccine in clinical trials using endogenous complement hSBA (enc-hSBA) assay


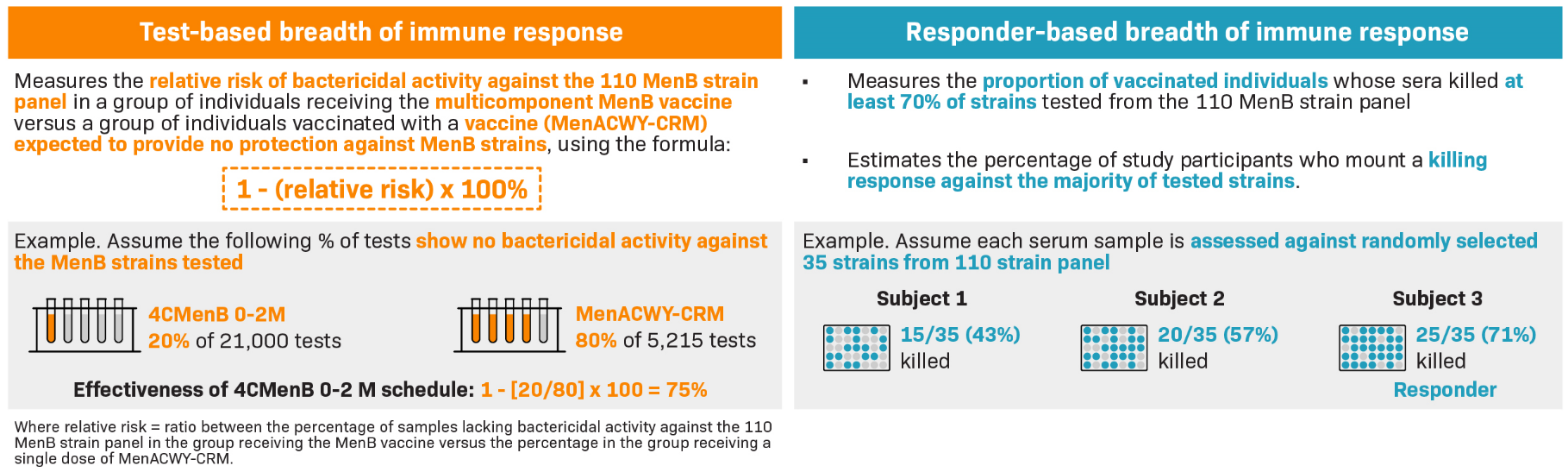


4CMenB, 4-component meningococcal serogroup B vaccine; hSBA, human serum bactericidal antibody; M, study month; MenACWY-CRM, meningococcal serogroups ACWY vaccine

## References

1. Borrow, R. et al. Methods to evaluate serogroup B meningococcal vaccines: From predictions to real-world evidence. *J. Infect.* **81**, 862-872 (2020).

2. Kleinschmidt, A., Vadivelu, K., Serino, L., Neidig, N. & de Wergifosse, B. Endogenous complement human serum bactericidal assay (enc-hSBA) for vaccine effectiveness assessments against meningococcal serogroup B. *NPJ Vaccines* **6**, 29 (2021).
